# Financing equitable access to antiretroviral treatment in South Africa

**DOI:** 10.1186/1472-6963-10-S1-S2

**Published:** 2010-07-02

**Authors:** Susan Cleary, Di McIntyre

**Affiliations:** 1 Health Economics Unit, School of Public Health and Family Medicine, University of Cape Town, Cape Town, South Africa

## Abstract

**Background:**

While South Africa spends approximately 7.4% of GDP on healthcare, only 43% of these funds are spent in the public system, which is tasked with the provision of care to the majority of the population including a large proportion of those in need of antiretroviral treatment (ART). South Africa is currently debating the introduction of a National Health Insurance (NHI) system. Because such a universal health system could mean increased public healthcare funding and improved access to human resources, it could improve the sustainability of ART provision. This paper considers the minimum resources that would be required to achieve the proposed universal health system and contrasts these with the costs of scaled up access to ART between 2010 and 2020.

**Methods:**

The costs of ART and universal coverage (UC) are assessed through multiplying unit costs, utilization and estimates of the population in need during each year of the planning cycle. Costs are from the provider’s perspective reflected in real 2007 prices.

**Results:**

The annual costs of providing ART increase from US$1 billion in 2010 to US$3.6 billion in 2020. If increases in funding to public healthcare only keep pace with projected real GDP growth, then close to 30% of these resources would be required for ART by 2020. However, an increase in the public healthcare resource envelope from 3.2% to 5%-6% of GDP would be sufficient to finance both ART and other services under a universal system (if based on a largely public sector model) and the annual costs of ART would not exceed 15% of the universal health system budget.

**Conclusions:**

Responding to the HIV-epidemic is one of the many challenges currently facing South Africa. Whether this response becomes a “resource for democracy” or whether it undermines social cohesiveness within poor communities and between rich and poor communities will be partially determined by the steps that are taken during the next ten years. While the introduction of a universal system will be complex, it could generate a health system responsive to the needs of all South Africans.

## Introduction

As the country with the highest number of HIV-infected people – accounting for a total of 17% of the global HIV burden [1] – treatment for HIV/AIDS in South Africa is a classic example of resource allocation in the face of highly constrained budgets. A key issue is that every treatment option for HIV has a large opportunity cost, particularly if the treatment strategy intends to provide coverage for a high percentage of those in need. This is partly because of the scale of the epidemic, and partly because HIV/AIDS is a new burden of disease. The allocation of resources to HIV-treatment is therefore not just about changing the scale at which the HIV-treatment programme is operating, but also about the creation of a new healthcare programme with associated training of health personnel, and investments in infrastructure, medical equipment, drug procurement and delivery systems.

In the recent “HIV&AIDS and STI National Strategic Plan” [2] the South African government committed to providing “an appropriate package of treatment, care and support services to 80 per cent of people living with HIV… by 2011” (p. 64). This “appropriate package” was defined, following the most recent “National Antiretroviral Treatment Guidelines” [3] to mean that antiretroviral treatment (ART) should be started (i.e. is needed) in adults once their CD4 count has fallen below 200 cells/μl and/or they have an AIDS diagnosis. The commitments contained within the national strategic plan are also argued to be guided by a number of principles including “tackling inequality and poverty” and “promoting equality for women and girls” (p. 60). Although no explicit equity goal is stated, the focus on increasing and sustaining high coverage could be argued to relate to horizontal equity – the equal treatment of equals – while the latter focus on guiding principles could be interpreted as ensuring vertical equity – the unequal but equitable treatment of unequals [4]. To date, there are around 700,000 people who have started on ART in South Africa [5] which means that this is by far the world’s largest programme. During 2008, just over 40% of new need was met.

South Africa’s health system includes both public and private financing and delivery. ART has been available in the private sector since as early as 1998, primarily via disease management programmes in private health insurance schemes (known locally as medical schemes). By 2004, when public sector ART provision began, well over 70% of those on ART in the country were in private care. However, by 2008 the situation had reversed with over 80% treated in the public sector [6,7]. This trend is likely to continue, with the implication that the public sector faces the majority of the HIV-related disease burden, with potentially major repercussions for affordability [8].

In terms of the overall resources available, the respectable 7.4% of GDP spent on healthcare in 2008 masks a highly unequal distribution of resources between the public and private healthcare sectors. Approximately 3.2% of GDP is spent within the public sector where the majority poor access care to the value of US$258 per person per annum. About 84% of the population is entirely dependent on the public sector for specialist and inpatient care. The other 16% of the population is covered by voluntary private health insurance and uses private sector services to the value of $1,363 per person per annum [Updated from: 9]. A further 1% of GDP is spent out-of-pocket on private sector providers, both in the form of co-payments by the insured and direct payments to private primary care providers (general practitioners and retail pharmacies) by a limited number of the uninsured. In addition, while substantial donor funding is available to support the scaling-up of ART, this amounts to only approximately 2% of the overall resource envelope available in the public health system (personal communication, Dr Mark Blecher, National Treasury South Africa).

Although funding for HIV-treatment has increased dramatically in the past 5 years in the public health sector, a programme of ambitious budget deficit reductions combined with personal income tax rate reductions has meant that the overall funding levels in the public health sector have been relatively stagnant until recently [10]. Additional file 1 illustrates how real per capita spending levels in the public sector actually declined from 1996 and only returned to 1996 levels in 2005, while spending via private voluntary health insurance increased rapidly in real terms leading to an increase in the public-private mix gap from a three-fold to more than a five-fold difference between 1996 and 2008. Service delivery in the public sector is increasingly hampered by large human resource shortages. Trends in human resources followed a similar pattern to real expenditure, with total staffing in the public health sector declining from about 251,000 in 1997 to about 215,000 in the early 2000s and returning to 251,000 by 2007/08. A recent report estimated that an additional 64,000 staff were required in 2007/08 to keep pace with the growth in the population dependent on the public health sector while 80,000 were required to accommodate both population growth and the growth in the burden of disease – largely related to HIV/AIDS [11].

There is growing consensus that the public health sector is inadequately resourced and there appears to be a commitment to gradually increasing public funding of health services to closer to 5% of GDP [11]. The context within which this is envisaged to happen is the introduction of a so-called ‘National Health Insurance (NHI)’, which the ruling party – the African National Congress (ANC) – committed itself to at its 2007 Polokwane Policy Conference and again during the 2009 elections. The ANC has been working on proposals for a NHI ‘behind closed doors’ and to date, no formal proposals have been put forward by government. Thus, the likely NHI design is unknown at this point. However, limited available information indicates that the broad vision is to focus energies primarily on rebuilding the public health sector to the point that it once again becomes the provider of choice of the vast majority of South Africans. This would be achieved by gradually, but substantially, increasing allocations from general tax revenue to the health sector as well as possibly introducing a compulsory NHI contribution for all formal sector employees. These funds would be pooled to promote access to publicly-funded health services that benefit all South Africans.  It has been proposed that the NHI would purchase services for the population from accredited healthcare providers, which could potentially include both public and private sector providers. It appears that the existing private voluntary health insurance schemes would continue, but may diminish in size over time.

The precise nature of future health system change is still unclear and will undoubtedly be influenced by the preferences of powerful stakeholders. There is also debate about whether the term NHI appropriately reflects the health system change that is envisaged for South Africa. What is clear though is that there is government commitment to achieving *universal coverage*. This objective is internationally supported, as demonstrated by the 2005 World Health Assembly resolution calling on member states to pursue universal coverage in their health systems [12]. The core elements of the concept of universal coverage are to provide financial protection against healthcare costs as well as ensuring access to quality healthcare for all when needed. In this paper, we prefer to refer to proposed health system change in South Africa as universal coverage (UC) reform. This is not only because the precise design of health system reform is yet to be finalised but also because, while we strongly support the goal of moving towards a universal health system, the term NHI frequently conjures up preconceived notions of a very specific health insurance model. We recognise that there are different ways in which UC can be achieved and that progress towards this goal must be appropriate within the South African context.

To date, most countries that have faced a comparable magnitude of AIDS epidemic, either in terms of the number of HIV-infected people such as India or HIV prevalence such as Swaziland, Botswana, Lesotho, Zimbabwe and Namibia [1] have relied heavily on donor funding for scaling up access to ART. A particularly striking example is that of Botswana, which received considerable external resources and technical support in scaling up equitable access to ART in a relatively short period of time. However, some countries cannot rely on external or donor support to the same extent. For example, South Africa is not regarded as a priority country for donor grants due to its relatively high level of economic development, while Zimbabwe has faced a massive decline in donor funding in protest against Mugabe’s rule. Zimbabwe has attempted to generate additional domestic funding through an AIDS levy, of 3% additional personal and company income tax [13]. South Africa is considering introducing UC reform with the aim of achieving universal health service coverage, including ART services, based on domestic public funding.

While donor grants will continue to be a critical funding source for health systems in many low- and middle-income countries for the foreseeable future, particularly in terms of funding AIDS interventions, there is a growing awareness of the need to expand domestic funding for health services. For example, the commitment by African Heads of State at a meeting in Abuja in 2001 to devote a minimum of 15% of government funds to the health sector was explicitly taken in recognition of the need to increase government spending on health services in order to address the massive burden of HIV/AIDS, TB and other infectious diseases facing countries in Africa [14]. More recently, the “Taskforce on Innovative International Financing for Health Systems” [15] has pointed to the need for more domestic resources, reduction in fragmentation of funding flows as well as an overall focus on health systems’ strengthening in order to achieve the health Millennium Development Goals (MDGs) which include the scaling up of HIV-treatment.

This consideration of funding scaled-up AIDS interventions in South Africa in the context of increased public domestic funding through the proposed UC reform is therefore potentially of considerable relevance to other countries which face a heavy AIDS burden and are seeking to respond to the World Health Assembly’s call for universal health service coverage through pre-payment funding mechanisms [16]. Given this background, the objective of this paper is to explore the affordability of achieving high coverage of those in need of ART in South Africa by and beyond 2020 within the context of the proposed UC reform.

In the remainder of the paper, two scenarios are modeled. In the first, the costs of scaled up access to ART are contextualized relative to a status quo scenario that assumes that the real growth in public healthcare resources keeps pace with projections of real growth in GDP. In the second scenario, the costs of scaled up access to ART as well as the costs of an adequately funded UC system are calculated. In what follows, the methods section outlines the data that have been used to estimate the costs of scaling up ART and the resource requirements for the UC system. Total and annual costs of ART are presented in the results section and compared to the status quo scenario and to proposed expenditure levels required within a universal health system. The final section includes a discussion and conclusions.

## Methods

### Costs of scaling up ART

#### Setting

The cost, utilization and outcomes data that inform this costing have been derived from the long-term follow-up of a cohort of adult HIV-positive patients receiving care in the sub-district of Khayelitsha, a largely informal area on the outskirts of Cape Town. In April 2000, three HIV clinics were opened in public facilities to provide treatment and prophylaxis of HIV-related and opportunistic infections and events, counselling and support groups for HIV-positive people. Acute infections were managed at the clinics while suspected tuberculosis (TB) cases were referred to TB facilities and severely ill patients to secondary and tertiary hospitals. In May 2001, the service was extended to include ART. After starting ART, patients continued to receive treatment and prophylaxis for acute infections as well as appropriate referrals for TB and hospital services.

#### Costing

The costing takes a public healthcare perspective in the context of scaling-up a large new healthcare programme over the long-run. Scaling-up requires medicines, laboratory investigations and other variable resources as well as long-run investments aimed at increasing the capacity of the healthcare system in order to provide the envisaged quantity of care while at the same time avoiding the crowding out of other priorities. The scope of costs therefore includes both variable and fixed direct healthcare costs given that both have an opportunity cost in this context. These costs were established for a full range of HIV-related services including HIV clinic visits and associated adherence support services, TB treatment and both district and tertiary level inpatient care. Given that unit costs within facilities can vary for a number of reasons including potential economies or diseconomies of scale, unit costs were calculated by pooling primary and secondary data from full economic cost analyses of four inpatient facilities, four ART clinics and four TB facilities [17-21]. Public sector ARV costs (including delivery costs to provincial depots) were sourced from the South African national ARV tender [22] and laboratory investigation costs were from the National Health Laboratory Services. In line with South African ART guidelines [3], patients receive stavudine, lamivudine and nevirapine or efavirenz as the first-line regimen. In the first and second-line treatment scenario, a patient failing the first-line regimen receives zidovudine, didanosine and lopinavir/ritonavir as second-line. CD4 and viral load monitoring occurs about twice a year.

#### Healthcare utilisation

HIV clinic, laboratory and ARV utilisation was calculated from 1,729 patients with 2,229 ART patient years of follow-up over a median follow-up of 1.03 years (IQR 0.68 – 1.70, max 4.08). Data on the use of inpatient and tuberculosis care required extensive validation. This was undertaken on 670 patients, with 693 ART patient-years. Relatively high mortality in the early months of ART and high rates of healthcare utilisation decreased once immune recovery had been achieved. This variability was captured directly from primary data. On the other hand, healthcare utilisation could be expected to increase for patients failing ART and dying. This was captured through specifying a “cost of dying”, based on a sample of 81 patients on ART who died of HIV-related causes.

#### ART model

Markov modelling was used to extrapolate primary data and to calculate the per-patient lifetime costs and outcomes of treatment and the total costs of treating all patients in need over the planning period. Separate Markov states were specified for CD4 50-199 cells/μl, and CD4 <50 cells/μl because these categories have been shown to be associated with different mortality rates in large cohort analyses [23,24]. Further heterogeneity between patients was observed to be related to the amount of time the patient had been on ART – mortality rates decreased steadily from the time of starting ART until the end of the follow-up period of 48 months. Similarly, the costs of healthcare are much higher in the first year because patients are relatively ill and could require inpatient care or TB treatment, are undergoing frequent laboratory investigations as specified by South African “National Antiretroviral Treatment Guidelines” (2004) and require more frequent visits to the clinic.

To capture this heterogeneity, the CD4-based Markov states were split into a number of temporary states, known as tunnel states. The use of these allowed the transition probabilities and costs to be varied as time on ART increased. During the first 6 month period, temporary states were created for each Markov cycle and for each CD4 category. After 6 months on ART, there was no longer any significant difference in costs and outcomes within CD4 strata, and these states were merged. However, differences in mortality probabilities and costs in relation to duration on ART were still significant, necessitating the ongoing use of tunnel states between 6 and 48 months on treatment. After 6 months on ART, the model included a probability of failing the first-line regimen. If first-line treatment was failed, the patient transitioned to the second-line regimen – the inclusion of separate states for the second-line regimen is required to capture the higher costs of these ARVs.

#### Transition probabilities

Transition probabilities are required to specify all relevant movements between Markov states In the ART models, these probabilities were estimated from Kaplan Meier product limit estimates of survival for the same 1,729 patients receiving ART in the first 48 months of the Khayelitsha programme, and extrapolated thereafter. The probability of dying was calculated directly from primary data and specified separately for each three-month cycle over the first 6 months on treatment, per 6-month period for months 6-12 and per annual period in months 12-24, 24-36 and 36-48 (no patients failed first-line in the first six months). This allowed an accurate specification of the decline in mortality over four years on ART. Patients who were lost to follow-up were conservatively treated statistically as deaths. The probability of transitioning to the second-line Markov states was calculated from primary data separately for months 6-12, 12-24, 24-36 and 36-48. We extrapolated death and failure transition probabilities conservatively by averaging over the full period of follow-up – this achieves a lower life expectancy than if we had extrapolated from later periods where far fewer deaths occurred. Because life-expectancy might be higher in pilot settings, the potential overestimate of mortality would increase the generalizability of results to routine services.

Additional details including unit costs, utilization estimates, transition probabilities

And full validations of the model including extensive multi-way and probabilistic sensitivity analyses have been published elsewhere [25].

#### Estimating the costs of scaling-up ART

Calculating the costs of scaling-up is achieved by entering estimates of total patient numbers into the model described above during each cycle of the planning period. The planning period is defined to start in 2004 which is when public sector ART provision began in South Africa. While the focus of this article is on the period from 2010 to 2020, the costs for patients who started ART prior to this time who are still surviving and remaining in care need to be included. Costs were therefore based on the *actual* patient coverage achieved from 2004 to 2008, patient coverage targets contained in the government’s strategic plan from 2009 to 2011 and after 2011, it has been assumed that the treatment coverage target reached by that date would be maintained until 2020. This is justifiable given that the 2011 target was to reach full coverage of those in need. Under the assumption that the government intends to fully fund their HIV-treatment plan, then the results of this costing exercise give an indication of the likely budget or resource envelope available for HIV-treatment over the period of analysis. These estimates of patients in need after 2012 have been extracted from the ASSA2003*lite* model (the leading model used in South Africa to estimate the total population with and without HIV/AIDS. These data were derived from ASSA2003*lite* (release 060315) as downloaded from http://www.actuarialsociety.org.za in May 2009). Full details of the assumptions of this model are available [26,27].

Costs were expressed in 2007 prices, converted to US dollars using an average 2007 exchange rate (US$1=7.05 South African Rands) [28]. Inflation adjustments were made using the consumer price index excluding mortgage bonds [29].

### Costs of UC

The modeling of the resource requirements for a UC health system draws on the SHIELD (Strategies for Health Insurance for Equity in Less Developed countries) project. This project is considering a range of scenarios for health system change to address existing inequities. The full range of scenarios, and extensive sensitivity analyses for each scenario will soon be published in full. The model presented here reflects what could be termed a basic universal coverage scenario, based on improved resourcing of public sector health services. It can be regarded as the minimum resourcing levels required for achieving universal coverage. This is appropriate to use in this paper as the purpose of this paper is *not* to critically evaluate alternative approaches to achieving universal coverage, but to illustrate the relative burden of ART costs within a universal health system context. Thus, adopting a relatively conservative approach to universal coverage resource requirements is appropriate. The main assumptions and methods for this model are described in detail in additional file 2.

There are three key variables in the model, which can be described by the following equation (with each of the three main variables disaggregated in various ways):

Total expenditure = population **x** service utilisation rates **x** unit costs

As there are considerable differences in utilisation rates between different age/sex groups, population and utilisation data are disaggregated into the following age groups: 0-4, 5-14, 15-49, 50-59 and 60+ years, expressed separately for females and males.

This approach is in line with the standard international approach to modelling future resource requirements with health system change, which is described as follows:

*“The general approach would be to assume that persons of different age groups would use a certain amount of services over a given period, and also to make certain assumptions regarding the development of prices over time. The multiplication of these utilization rates and unit costs yields expenditure for each type of service analysed. These expenditure sub-totals are then added to arrive at total benefit expenditure with the system” *[30].

Utilisation rates are also disaggregated for different categories of services (primary healthcare visits and in- and out-patient care at district, regional and tertiary or central hospitals) and are specified as the number of visits per person per year and number of inpatient days per person per year. The unit costs for each of these different services are specified as cost per visit and cost per inpatient day.

The baseline age/sex utilisation rates for each type of service were derived from a national household survey conducted in 2008 which focussed exclusively on collecting health and health service related information [31].

Current utilisation rates of public sector health services are relatively low in South Africa. A number of detailed studies have been undertaken in the past which have carefully calculated utilisation rate norms based on a range of factors, including burden of disease and drawing on international experience of health service utilisation in well funded health systems. These studies were drawn on to estimate future utilisation rates. In the case of primary healthcare services, the ‘Need Norms’ study undertaken by the Centre for Health Policy [32] was used, but was updated to account for increased service requirements for those with HIV/AIDS before they are eligible for ART. In the case of in- and out-patient hospital services, the utilisation rates recommended by the ‘Hospital Strategy Project’ were used [33]. It was assumed that these utilisation levels would be fully phased in by 2020.

Current unit costs were calculated by combining information on expenditure in each public hospital in South Africa (sourced from National Treasury) with information on the number of inpatient days and outpatient visits at each hospital (sourced from the Health Information System database maintained by the national Department of Health).

As indicated previously, the public health system is currently under-resourced with real expenditure and staffing levels not keeping pace with the growth in the population dependent on public sector services (or with the growing burden of disease in South Africa). A recent study estimated that an additional US$3.3 billion is required to redress this resource shortfall, particularly in relation to dramatically improving staffing levels to, as a minimum, at least return to the staff-to-population ratios that prevailed in the mid-1990s [11]. This amount was used to calculate the increase in unit costs that would be required to achieve these quality improvements prior to any increase in utilisation. This increase in unit costs was phased in by 2015, whereafter real unit costs are assumed to only increase by 1% per annum (i.e. further expenditure increases would be driven largely by increases in utilisation rates).

The national household survey did not provide adequate data on the use of specialised hospitals (psychiatric, TB etc. hospitals). For this reason, a lump-sum amount was added for these services, based on current expenditure on these hospitals with increased resourcing comparable to that outlined above for other health services. Other services such as community-based services, emergency medical services (ambulances etc.) and training of health professionals were also added on to the total resource requirements projected by the model. In addition, additional administration costs were included.

The ASSA2003 projections of population growth, disaggregated using the same age/sex categories as for utilisation, were used. We assumed that while the entire population would be eligible for benefits from the UC system, a portion of the population will choose to maintain private health insurance and only draw on 25% of the benefits of the UC system.

#### Estimates of GDP growth

We have estimated GDP growth over the period based on National Treasury’s assessment of the economic outlook in early 2010 [34], with a conservative extrapolation into the future. Treasury estimated that real GDP growth was 3.7% in 2008 and -1.8% in 2009 and projected it would increase by 2.3% in 2010, 3.2% in 2011 and 3.6% in 2012. The assumed real GDP growth rate in future is:

➤ 4% in 2013 and 2014; and

➤ 4.5% from 2015 to 2020

These growth rates are considerably lower than the real GDP growth rates of 5% or above experienced between 2005 and 2007 (National Treasury 2009).

All ART costs, UC resource requirements and GDP estimates are presented in real terms in 2007 US$.

## Results

### ART annual and total resource needs

If high coverage of ART is achieved and sustained, the annual amount required is expected to increase from US$1 billion in 2010 to US$3.6 billion in 2020, which is close to a three-fold increase in total spending across the period. The 43% increase needed from 2009 to 2010 steadily decreases over the period to stabilize at a level of around 6% per annum by 2020. During the same period, the number of patients surviving and remaining in care on ART is estimated to rise from 1 million to 3 million. These results are summarized in additional file 3.

### ART resource needs as a proportion of the current public health system

During the calendar year of 2007, approximately US$8.7 billion was spent in the public healthcare system [35], of which around 4% would have been required to support the ART programme and related care (inpatient care, treatment for tuberculosis etc). If high coverage of those in need of ART is maintained and if the growth in funding to the public healthcare system keeps pace with projected real growth in GDP, then close to 30% of these resources would be required for ART by 2020 (see additional file 4).

### Annual resource requirements for UC

In order to redress the substantial under-resourcing of the public health sector over the last decade and a half, public funding as part of the UC reforms (excluding resource requirements for those on ART) will need to increase rapidly until 2015, by which time expenditure of approximately US$17.6 billion would be needed. Thereafter, resource requirements will increase more gradually, largely related to utilization increases, until a level of US$22 billion is reached in 2020.

### ART resource needs within the context of UC

The UC scenario, including ART costs, modeled in this paper requires more than a doubling of total system expenditure in real terms from 2010 to 2020. Increases are phased in rapidly initially, with 12% higher real expenditure in 2011 versus 2010. These increases decrease over the period, to reach around 5% by 2016 and 4% by 2020. ART expenditure is 12.7% of total expenditure over the period, ranging from nearly 9% in 2010 to slightly over 14% in 2020. Total annual expenditure will be approximately US$25.6 billion by 2020. See additional file 5.

Additional file 6 illustrates the implications of this level of public funding of health services, including the costs of those on ART. Expenditure on the UC system will need to be about 4% of GDP by 2010 and increase rapidly to 5.6% of GDP by 2015 and more gradually thereafter to 5.7% of GDP by 2020.

## Discussion

This paper has examined the overall costs of scaling up access to ART from 2010 to 2020 within the context of the proposed UC system. Coverage has been defined following the National Strategic Plan for HIV&AIDS and STIs to be meeting 80% of new need for ART on an ongoing basis. The costing of ART is subject to a number of key uncertainties including those related to the data requirements of the study, extrapolation of data and generalizability of results. Uncertainty relating to data requirements has previously been assessed using probabilistic sensitivity analysis (PSA) while generalizability has been assessed via multi-way sensitivity analyses [25]. While these analyses lend some support to the overall robustness of the lifetime costs of care used in this current analysis, it is nevertheless the case that this costing is based on a relatively efficient model where care is delivered via community health centers and treatment is handled by both doctors and nurses. This model of care is cheaper than a number of alternatives that have been evaluated in the South African public [36] and private healthcare sectors [37]. It should therefore be borne in mind that the ART costs presented in this paper could be underestimated, given that a scaled up response is likely to require delivery of ART through all possible models of care. It is also likely that costs would increase following any decision to change the CD4 initiation criteria from <200 to <350 cells/μl. While starting ART at CD4<350 cells/μl will be likely to be associated with lower costs during the first year on treatment [37], cost-effectiveness analyses have shown that lifetime costs would increase, primarily owing to higher life expectancy [38,39]. There is an urgent need to model the implications of this change, which would require data on lifetime costs from a generalizable model of care, estimates of numbers in need as well as an indication of the likely demand for this care under different extensive HIV-testing scenarios. On the other hand, if the steady decrease in ARV prices of the last decade is maintained and the exchange rate depreciations resulting from the financial crisis abate, then some of the costs could be overestimated in this paper.

Two scenarios have been explored in this paper. In the first, resources in the public healthcare system are assumed to stay constant in real terms at the 2007 level. Under this scenario, scaling up access to ART would consume 27% of these resources by 2020. In the second scenario, the resources required to provide ART were contrasted against the overall public resources available if a UC health system were implemented, noting that only one scenario is presented here which is simply based on the resource requirements to improve the quality of public sector services and increase access to and utilization of these services. The proportion of total expenditure on ART would be a maximum of just over 14% in 2020 and an average of 12.7% over the period. This seems a far more feasible challenge and would be accompanied by measures to redress historical inequities in the overall health system.

We acknowledge that: *“A model is not a crystal ball; it does not predict the future. Rather, models project a possible future state on the basis of observations and assumptions on future conditions.” *[30]. The UC scenario we have presented here is only one of a series of scenarios we have developed, and it is highly dependent on the assumptions made (which are spelt out in the technical annex). It represents what could be considered the minimum resource requirements for a universal health system which provides both financial protection and access to needed health services for all. The focus of this paper is not to consider in detail alternative ways of achieving universal coverage in South Africa and associated resource requirements. Instead, the intention is to illustrate how different the challenge of scaling-up ART is in the context of currently under-resourced public health services compared to a context of a commitment to improving the domestic public funding of health services with a goal of achieving universal coverage.

There will be a number of challenges in implementing UC reform in South Africa. The extent to which the allocation from general tax revenue to the health sector can grow, without increasing income tax rates, is dependent on continuing to achieve strong economic growth. One potential source of increasing tax revenue is the proposed removal of tax deductibility of medical scheme contributions, the value of which was estimated to be almost US$ 2 billion in 2007 [40]. There will be a need for careful phasing in of the changes to ensure that the absorptive capacity of the health system is not exceeded. Importantly, there will undoubtedly be opposition from key interest groups, particularly private insurance groups and providers and the highest income earners. International experience demonstrates that the policy process, particularly in dealing with such opposition, will require careful management [41-43]. In addition, careful design of the universal coverage system is required to ensure that it is affordable and sustainable within the South African context. For example, high quality services at the primary care level and stringent gatekeeping to specialist and hospital based services are essential, as are appropriate provider reimbursement methods (e.g. capitation with global budget caps if primary care services are purchased from private providers).

Despite these challenges, the goal of sustaining ART access through achieving public funding levels approaching 5-6% of GDP, whether solely through general tax allocations or from general tax complemented by a mandatory health insurance contribution (or dedicated health tax), is within the realms of what would be regarded internationally as affordable and sustainable. While many of the countries with expenditure levels of 5% of GDP or more within the public healthcare sector are in the high-income country category, there are a growing number of middle-income (and some low-income) countries that have achieved these spending levels. For example, Brazil has public funding for healthcare of 4.8% of GDP, Costa Rica a level of 5.1%, Cuba 5.5% and Colombia 6.7% of GDP [44]. In all of these countries, there has been a strong commitment to public funding of social services and striving to achieve a universal health system.

The analysis presented here indicates that the challenge of achieving high coverage of ART in South Africa within existing public sector resource levels could be quite overwhelming in countries with a large HIV-epidemic. There is considerable potential for HIV interventions to adversely impact on other needed health services, given the urgency and magnitude of the need for such interventions. However, if scaling up ART access is considered within the context of overall health system reform to achieve universal coverage, the challenge becomes less daunting. Thus, there is value in also focusing policy attention on moving towards universal health system coverage rather than focusing exclusively on the resource requirements for scaling-up ART access. While there is an urgent need to meet the treatment needs related to the HIV-epidemic (and other communicable disease epidemics), there is an equally urgent need to give more attention to moving towards universal health systems.

The international experience of moving towards universal cover in high-income countries and middle-income countries in Asia and Latin America does not provide any insights into how this can be achieved within the context of a large burden of HIV/AIDS. Therefore, the proposed UC reform in South Africa provides an important opportunity for monitoring and evaluating efforts to meet the emergency of the HIV-epidemic in the context of achieving a universal health system.

What we do know from international experience is that universal coverage can best be achieved through having an *integrated* pool of public funds [45]. What is urgently required in low- and middle-income – mainly African – countries facing a large HIV-epidemic is to focus attention on integrating public funds (whether from general tax revenue, mandatory health insurance contributions or donor sources) into a single pool and to increasing these funds, particularly from domestic sources, over time. Progress towards achieving the Abuja target of 15% of general tax funds being devoted to the health sector has been extremely limited [46]. This must be addressed, and other options such as dedicated health taxes or mandatory health insurance contributions considered in order to gradually increase *domestic public funding* for health services. This is important as donor funding can be unreliable and irregular, sometimes varying considerably from year to year, and is unlikely to be sustained at current levels in the long-term. In addition, donor funding to African countries is heavily focused on the key communicable diseases of AIDS, TB and Malaria. Universal health systems are unlikely to be feasible without increased domestic public funding, particularly if the goal of universalism is being pursued at the same time as scaling up ART interventions. It is only when there is increased policy attention on achieving a universal health system that equitable ART access will be affordable and sustainable and will not compromise the goal of equitable access to other health services.

## Conclusion

Responding to the HIV-epidemic is among one of the many challenges currently facing the new South African democracy. Whether this response becomes a “resource for democracy” [47 p. 497] or whether it undermines social cohesiveness between rich and poor communities will be partially determined by the steps that are taken during the next ten years. Mooney [48] argues that a country’s healthcare system is a reflection of the values of its citizens. If this is the case, a successful response to the HIV-epidemic within a health system that is funded from a substantial and integrated pool of public funds could contribute to moving from a past marked by highly unequal access to freedom and resources to a future characterized by cohesiveness and solidarity.

## Competing interests

The authors declare that they have no competing interests.

## Authors’ contributions

SC calculated the costs of ART; DM calculated the costs of UC. Both authors contributed to the writing of the paper and agreed with the manuscript’s results and conclusions.

## Supplementary Material

Additional file 1 Trends in real per capita spending in the public and private health sectors (Real terms; Base year = 2008) Click here for file

Additional file 2 Technical Annex Click here for file

Additional file 3 Annual ART costs (in US$ 1,000’s) and total patients surviving on ART (expressed in 2007 US$) Click here for file

Additional file 4 Annual ART costs as a proportion of “status quo” public health spending (expressed in 2007 US$) Click here for file

Additional file 5 Annual UC and ART costs (expressed in 2007 US$) Click here for file

Additional file 6 Annual UC and ART costs as a percentage of GDP Click here for file
