# Supplementary material for: Financing equitable access to antiretroviral treatment in South Africa
Source: BMC Health Serv Res. 2010 Jul 2;10(Suppl 1):S2. doi: 10.1186/1472-6963-10-S1-S2 (PMC2895746; doi:10.1186/1472-6963-10-S1-S2)
Supplement: Additional file 1 — Trends in real per capita spending in the public and private health sectors (Real terms; Base year = 2008) [file 1472-6963-10-S1-S2-S1.docx]

Trends in real per capita spending in the public and private health sectors (Real terms; Base year = 2008)


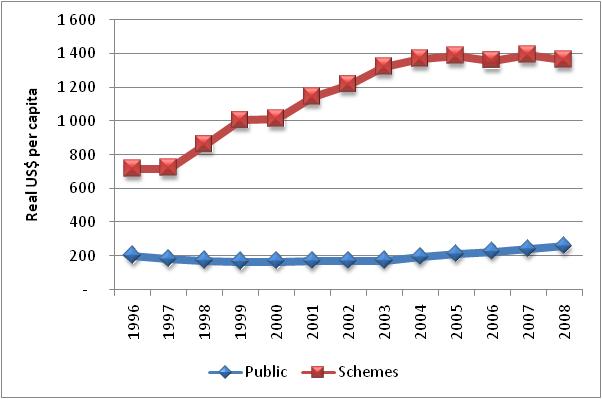


**Source: Updated from: [49]**
