# Supplementary material for: Financing equitable access to antiretroviral treatment in South Africa
Source: BMC Health Serv Res. 2010 Jul 2;10(Suppl 1):S2. doi: 10.1186/1472-6963-10-S1-S2 (PMC2895746; doi:10.1186/1472-6963-10-S1-S2)
Supplement: Additional file 2 — Technical Annex [file 1472-6963-10-S1-S2-S2.docx]

**TECHNICAL ANNEX**

**Detailed methodology for modeling the resource requirements for UC**

This is an admittedly simple model designed to produce ‘high-level’ estimates of the resource requirements for a universal health system under different conditions. There are undoubtedly areas where further refinement is possible. Only one scenario is presented in this paper; a wide range of scenarios and sensitivity analyses, along with more extensive methodological details, will be published elsewhere. The model presented here can be considered to be an estimate of the minimum resource requirements for achieving universal coverage in South Africa.

Basic approach

The model is structured to answer the question: “what will be the total expenditure on healthcare in a universal health system” or “what financial resources will be required for health services under a universal health system”. There are three key variables in the model, which can be described by the following equation (with each of the three main variables disaggregated in various ways):

Total expenditure = population **x** service utilisation rates **x** unit costs

Population and utilisation data are disaggregated into the following age groups: 0-4, 5-14, 15-49, 50-59 and 60+ years, expressed separately for females and males, to account for differences in utilisation rates between different age/sex groups. Utilisation rates and unit cost data are also disaggregated according to type of health service. These health service categories are based on what currently exists within the health system, particularly: Primary care services, outpatient visits and inpatient days at district, regional and tertiary or central hospitals.

As various services are not included in the basic model, a lump sum amount is added to the output of the model to ensure that total resource requirements are captured. The services included in this lump sum are:

- Specialised hospitals for tuberculosis and psychiatric care
- Emergency medical services
- Community-based services
- Health worker training
- Healthcare support services
- Health facilities management
- Services falling under the national Department of Health
- Administration at all levels of the public health system

Before any projections were undertaken, the model was ‘benchmarked’ for the years 2007 and 2008. The total expenditure predicted by the model was compared to actual expenditure on these services as reported by Treasury and the Council for Medical Schemes. The expenditure predicted by the model was highly comparable to the actual expenditure recorded in official audited financial statements.

Population data

Population data are drawn from the Actuarial Society of South Africa (ASSA) dataset, more specifically the most recent version of the ASSA suite of models, the ASSA2003 AIDS and Demographic Model (downloaded from [www.assa.org.za](http://www.assa.org.za)). ASSA is regarded as the pre-eminent body undertaking demographic modelling of the South African HIV-epidemic, which is the factor with the greatest impact on demographic trends in South Africa at present. No alternative demographic projections are available that are regarded as reliable. For this reason, no sensitivity analyses using other demographic projection estimates were undertaken.

The population is divided into those covered by medical schemes (private voluntary health insurance) and those not covered by schemes. Even under a universal publicly funded health system, some of the wealthiest South Africans will choose to have private health insurance cover over and above being entitled to benefit from services that are publicly funded. Current medical scheme membership levels were derived from the relevant regulatory authority [50]. The potential impact of substantial health system change on medical scheme membership is a matter of speculation. In the full scenario modelling, we have used a range of assumptions. In the model presented here, we have assumed that medical scheme membership will decline by 40% from current levels over a 10 year period. The basis for this assumption is as follows: Medical scheme contributions have been increasing at rates far exceeding general inflation for several decades. Without substantial changes in the medical schemes industry, it is very unlikely that scheme membership will grow in the context of a relatively comprehensive package of services with substantially improved resourcing over current public sector services via a UC system. At present, 43% of scheme members pay contributions which are 10% or more of their income (based on an analysis of Statistics South Africa’s 2005 Income and Expenditure Survey); those contributing at this level may opt out of medical schemes in future. Those who are not covered by medical schemes are assumed to fully use services to which they are entitled while those who are covered by medical schemes are assumed to have paid for that cover so that they do not have to rely on their entitlements under the UC system and are therefore assumed to only use a maximum of a quarter of their UC service benefit entitlements.

Utilisation data

Potentially the most critical assumption in the model is that the ‘public sector framework’ of service delivery was assumed to be the most appropriate basis for modelling a future UC system. The rationale for this assumption is that the information available about the proposed ‘NHI’ indicates that:

*“There will be a comprehensive package of services that includes primary health care services as well as hospital inpatient and outpatient care. People will be expected to follow the appropriate referral route to ensure effective gate-keeping as at the primary health care level before referrals to specialists and hospital-based care when necessary. This will ensure that resources are used efficiently and appropriately.”* (ANC Today, 23-29 January 2009, Volume 9, Number 3)

This strongly implies that the benefit package covered under the UC system will be more similar to the current ‘public sector framework’ than the current ‘medical scheme framework’. While medical scheme packages use a very explicitly stated ‘positive list’ of specific services that will be covered (e.g. whether or not chronic medicines for depression are covered), the public sector currently provides a comprehensive package of services with an implicit ‘negative list’ (i.e. services that the public sector simply does not provide) and implicit rationing (e.g. a limited number of dialysis machines are available and clinicians have to select which patients should receive priority in being treated with these machines). This comprehensive package is defined in terms of individuals having access to primary care facilities, for which there are ‘norms’ in relation to the type of staff that should be employed, equipment that should be available and range of services that should be provided, and to specialist and hospital care on referral. There are also broadly specified ‘norms’ in terms of the range of services that should be available at the different levels of hospitals. This appears to be more in line with the benefit package model envisaged for the UC system.

Data on the current utilisation of different types of health services is drawn from a national household survey, called SACBIA (the South African Consortium for Benefit Incidence Analysis), which was initiated by the Health Economics Unit at the University of Cape Town, the Centre for Health Policy, University of the Witwatersrand, and the national Department of Health. Data collection was contracted to an experienced survey company, the Community Agency for Social Enquiry (CASE).

The survey was nationally representative. Enumerator areas (EAs) were stratified by province, type of settlement (farm, informal settlement, tribal settlement, small holding, and urban settlement) and population group. In total, 960 EAs were selected across the nine provinces and five randomly selected households were interviewed within each EA, giving a total sample size of 4,800 households. The EAs within each stratum were selected with a probability proportional to the size of the EA, defined as the number of households within it. Fieldworkers were given extensive training to ensure the questions were well understood. Data was collected in May and June 2008. Twenty percent of questionnaires were subjected to telephonic ‘check-backs’ for verification and double-entry data capture reduced errors. The data were weighted to national population levels. The questionnaire and study protocol were subject to ethical review by the University of Cape Town, and all respondents provided signed informed consent.

The recall period for outpatient visits in the SACBIA survey is one month and for inpatient admissions is one year, which is consistent with international practice [51]. Seasonality indices were applied to outpatient visit data to generate annual *utilisation rates*, taking account of seasonal variations in visits based on the month and the province the survey was conducted rather than the traditional fixed multiplication by 12 for a recall period of one month [51]. This is because of seasonal patterns in disease incidence as well as variations in the health seeking patterns of households [52].

Two primary sources of likely future utilisation rates were used, namely the ‘Need Norms’ study for primary care services [32] and the ‘Hospital Strategy Project’ (HSP) for out- and in-patient services in hospitals [33]. Both of these studies undertook very extensive reviews of ‘ideal’ utilisation levels given the demographic and epidemiological profile of the South African population. They were essentially trying to establish a set of ‘*norms*’ for health service utilisation. For example, the ‘Need Norms’ study took into account the number of primary care visits required to ensure that young children are fully immunised, the number of antenatal visits recommended for pregnant women, the incidence of tuberculosis and the number of visits required for diagnosis, treatment and monitoring of tuberculosis patients based on the standard treatment protocols etc. Both studies recognised that service uptake levels are seldom at the ‘ideal’ level (e.g. pregnant women in low- and middle-income countries often do not comply with the full number of antenatal care visits that are recommended), and came up with a set of what could be regarded as more ‘realistic’ utilisation norms. This model has used the ‘realistic’ utilisation norms, which still translate into quite large utilisation increases over current levels. An example of the utilisation rates are presented below in relation to district hospital care.

**District hospital service utilisation rates used in model**

| **Age/sex group** | **Utilisation of outpatient services: average visits per person per year** | | **Utilisation of inpatient services: average inpatient days per person per year** | |
| --- | --- | --- | --- | --- |
|  | **Current** | **Future** | **Current** | **Future** |
| Females 0-4 | 0.15 | 0.28 | 0.08 | 0.17 |
| Females 5-14 | 0.07 | 0.14 | 0.06 | 0.12 |
| Females 15-49 | 0.34 | 0.63 | 0.26 | 0.55 |
| Females 50-59 | 0.28 | 0.52 | 0.27 | 0.58 |
| Females 60+ | 0.48 | 0.89 | 0.46 | 0.97 |
| Males 0-4 | 0.17 | 0.32 | 0.11 | 0.24 |
| Males 5-14 | 0.08 | 0.14 | 0.07 | 0.15 |
| Males 15-49 | 0.13 | 0.25 | 0.14 | 0.29 |
| Males 50-59 | 0.24 | 0.45 | 0.24 | 0.52 |
| Males 60+ | 0.48 | 0.88 | 0.39 | 0.82 |

It was assumed that moving from current utilisation levels to the relatively higher utilisation levels would be achieved over a 10 year period. A key constraint to utilisation increases is existing service supply (facilities, personnel, etc.). It is unlikely that utilisation will increase more rapidly than supply will permit. There will undoubtedly be fluctuations in utilisation. For example, when free care at primary care level was introduced in South Africa, utilisation increased substantially and rapidly. This is likely to have been largely due to previously unmet need. However, within a few months, utilisation settled at a level that was higher than before the removal of fees, but lower than the initial spike. As supply increases and waiting times at facilities decline, utilisation will begin to increase further.

Unit cost data

To calculate the current unit costs of public sector services, data on recurrent expenditure for each type of facility (and for individual facilities in the case of hospitals) was obtained from National Treasury. In addition, total utilisation of public sector facilities was obtained from the National Department of Health’s District Health Information System (DHIS) dataset. In the case of clinics and community health centres, the unit cost was simply calculated as expenditure divided by number of visits. In the case of hospitals, unit costs are calculated as the total recurrent expenditure in a facility divided by the weighted total number of users (inpatients and outpatients). Frequently, the assumption made (even internationally) is that the cost of an outpatient visit is a fixed proportion of the cost of an inpatient day. An outpatient visit for instance is considered to be one-third or one-fourth of the cost of an inpatient day across all hospitals (Adam and Evans, 2006, Barnum and Kutzin, 1993, Lombard et al., 1991). However, this assumption is not based on a solid empirical basis and previous research has shown that the ratio of expenditure per inpatient day to expenditure per outpatient visit varies considerably across hospitals at different levels of care [53].

For this reason, we calculated the ratio of the unit costs for inpatient care and outpatient visits for different categories of public sector hospitals using statistical methods. Data on total outpatient and inpatient utilisation by type of hospital and by province was extracted from the DHIS, as well as the total number of nurses and medical doctors – as a proxy for the relative size of the facility. These data were combined with data on recurrent expenditure at each public sector hospital in South Africa, provided by the National Treasury. A simple regression model was fitted to generate the ratio of variation between the cost of an outpatient visit and an inpatient day across various public facilities. This ratio was then used to estimate the unit costs for inpatient and outpatient services for each hospital category.

The main assumptions in terms of unit costs in the model relate to what may happen to unit costs when a universal health system is pursued. There is general consensus that the public health system is currently under-resourced, particularly in terms of staffing levels. For this reason, as a minimum, it is necessary to assume that the current unit cost levels in the public sector are an underestimate of the resources required to provide an adequate range and quality of services. The Development Bank of South Africa (DBSA) undertook an extensive critical analysis of the existing resourcing of the health system and estimated the minimum resources necessary to get the public health system back to a position of being appropriately resourced [54]. The DBSA analysis was used to estimate the likely increase in unit costs, which was supplemented by accounting for the recent substantial salary increases for a range of health professionals working in the public health sector. The DBSA recommended that these resourcing improvements should be phased in over a 5 year period. These estimates translated into annual real increases of 6.8% in unit costs for the next five years. It was assumed that after this initial injection of resources, unit costs would continue to increase above inflation at about 1% real increase per year (to allow for more gradual but continued increases in resourcing levels.
