# Supplementary material for: Financing equitable access to antiretroviral treatment in South Africa
Source: BMC Health Serv Res. 2010 Jul 2;10(Suppl 1):S2. doi: 10.1186/1472-6963-10-S1-S2 (PMC2895746; doi:10.1186/1472-6963-10-S1-S2)
Supplement: Additional file 6 — Annual UC and ART costs as a percentage of GDP [file 1472-6963-10-S1-S2-S6.docx]

Annual UC and ART costs as a percentage of GDP
